# High-Frequency Vestibular Function Is Vulnerable to Presbyvestibulopathy

**DOI:** 10.3390/diagnostics14121224

**Published:** 2024-06-11

**Authors:** Seonghoon Bae, Jimin Yun, Seungmin Kwak, Hyuntaek Jung, Hancheol Lee, Juyoung Kim, Chanhee Kim, Yujin Lee, Sunghuhn Kim

**Affiliations:** 1 Department of Otorhinolaryngology, Gangnam Severance Hospital, Yonsei University College of Medicine, Seoul 06273, Republic of Korea; 2 Department of Otorhinolaryngology, Yonsei University College of Medicine, Seoul 03722, Republic of Korea

**Keywords:** aging, presbyvestibulopathy, vestibular function test, video head impulse test, caloric test

## Abstract

Introduction: In 2019, mild vestibular function deficiency in elder populations was defined as presbyvestibulopathy (PVP) by the Classification Committee of the Bárány Society. The diagnostic criteria include tests for low-, mid-, and high-frequency vestibular function, represented by caloric testing, rotary chair testing, and head impulse testing, respectively. However, there is still a lack of large-scale reports supporting the relationship between vestibular function tests (VFTs) and aging. In this study, we evaluated whether each test is correlated with aging in the elderly population aged over 50. Methods: This study retrospectively enrolled 1043 subjects from a single university hospital database after excluding those with unilateral and bilateral vestibulopathy, central dizziness, and acute dizziness. Enrolled subjects had caloric canal paresis <20%, vHIT lateral canal gain >0.6, vHIT interaural difference <0.3, and age >50 years old. Results: Significant negative correlations with age were identified in the vHIT (*p* < 0.001) and rotary chair test (RCT) 1.0 Hz gain (*p* = 0.030). However, the caloric test (*p* = 0.739 and 0.745 on the left and right sides, respectively) and RCT 0.12 Hz gain (*p* = 0.298) did not show a significant correlation with age. A total of 4.83% of subjects aged 70 years or older showed sub-normal vHIT gain that met the criteria of PVP, whereas only 0.50% of subjects aged 60 to 69 did. The prevalence of sub-normal caloric test results, however, was not significantly different between the two age groups (21.55% in the 60–69 age group and 26.59% in the >70 age group). Conclusions: The high-frequency range vestibular function seems vulnerable to aging, and this is more discernible at age >70 years. The weak correlation between age and low-frequency vestibular function tests, such as the caloric test and low-frequency rotary chair testing, suggests the need to revisit the diagnostic criteria for PVP.

## 1. Introduction

Dizziness and imbalance among the elderly are well-known escalating public health problems owing to their significant correlation with the risk of falling injuries, which can even result in death [1]. Dizziness and imbalance are common problems (24% of people older than 72 years in the United States) in the elderly; furthermore, its frequency steeply rises with age, affecting over 50% of the population over aged 80 [2]. Although there may be complex multifactorial reasons for these symptoms, vestibular dysfunction seems to play a significant role given its physiological importance in maintaining body balance [3]. Recently in 2019, vestibular function deficiency in these populations was defined as presbyvestibulopathy (PVP) by the Bárány Society classification committee [4].

The diagnostic criteria for PVP include not only typical symptoms, postural imbalance, gait disturbance, chronic dizziness, and recurrent falls, but also objective findings that present as bilateral sub-normal peripheral vestibular function. The laboratory tests for delineating the objective sub-normal vestibular function are the caloric test (maximum peak slow-phase velocity on each side 6–25°/s), video head impulse test (vHIT, lateral canal gain 0.6–0.8 bilaterally), and rotary chair test (RCT, 0.1 Hz of sinusoidal harmonic stimulation gain 0.1–0.3). In addition, each of the three diagnostic tests for evaluating lateral semicircular canal function covers different frequency ranges of vestibular stimulation: the caloric test represents very low-frequency stimuli (around 0.003 Hz), the rotary chair test (RCT) represents mid-frequency stimuli (about 0.01 to 1 Hz), and the video head impulse test (vHIT) represents higher-frequency stimuli (around 5 Hz) [5,6,7,8]. However, compared with vestibular-evoked myogenic potential (VEMP), which has plenty of evidence of age dependence, the correlation between aging and the three laboratory tests for diagnosing PVP has not been fully confirmed [9,10,11]. In addition, VEMP has been excluded from the diagnostic criteria because of its lack of standardization and mechanistic understanding, in contrast to tests such as the caloric, vHIT, and RCT [4,12].

In this study, we analyzed 1043 subjects aged >50 years who underwent the vestibular function test (VFT) in a single center using identical VFT settings. This study aimed to verify the diagnostic tests for PVP including caloric test, RCT, and vHIT, which respectively represent low-, mid-, and high-frequency vestibular function, are correlated with aging in the elderly population. Furthermore, this study sheds light on the aging process of the vestibular system and extends the discussion on the diagnosis of PVP.

## 2. Methods

### 2.1. Subject Enrollment

A total of 1946 subjects aged >50 years who underwent both the caloric test and vHIT between 1 January 2018 and 31 December 2021 were screened from the Severance Hospital (Seoul, Republic of Korea) database. The exclusion criteria were as follows: (1) 730 cases of unilateral vestibulopathy (caloric canal paresis (CP) ≥ 20 or vHIT lateral canal gain <0.6 or vHIT interaural difference >0.3 or RCT asymmetry more than 2 frequencies), (2) 35 of bilateral vestibulopathy (according to the criteria suggested by the Bárány Society in 2015), (3) 106 of caloric test failure (air irrigation was also excluded), (4) 30 of spontaneous nystagmus (any direction) or gaze-evoked nystagmus, (5) 1 of blindness in one eye, (6) 1 of vHIT failure, (7) 0 of central dizziness including head trauma, and (8) 0 of acute dizziness spell within 2 weeks before the tests. Consequently, 1043 subjects were included. The study protocol was approved by the Institutional Review Board of Severance Hospital (Project Number 3-2023-0363). The requirement for consent was waived by the same institution because of the retrospective design of the study.

### 2.2. Vestibular Function Tests

The bithermal caloric test was performed using an infrared video-oculographic system version 9.0 (Visual Eyes VNG; Micromedical Technologies, Chatham, IL, USA). Each ear canal was stimulated with closed-loop water irrigators at 30 °C and 44 °C for 30 s, with the patient in a supine position with 30 degrees of head flexion and had a resting interval of 5 min between irrigation cycles. During irrigation, induced nystagmus and slow-phase velocity (SPV) were monitored using a videonystagmography system. Unilateral weakness (canal paresis (CP)) was calculated using Jongkee’s formula [13]. The time window for SPV analysis was 10 to 90 s after irrigation, including the maximal slow-phase velocity period. Artifacts were initially excluded by the algorithm of the software and subsequently by manual review from the test inspector. The peak period of slow-phase eye movement was automatically selected by the software and then confirmed or modified through manual review by the test inspector.

A vHIT device from GN Otometrics (ICS Impulse; GN Otometrics, Taastrup, Denmark) was used to record eye movements in a two-dimensional plane. Default software settings were used. To evaluate the horizontal semicircular canals, the participants were seated upright with 30 degrees of head flexion and instructed to gaze at a dot on a wall at a distance of 1 m. The head impulses were conducted by the same right-handed examiner with a peak velocity range of 200–250 degrees/s, rotation amplitude of 15–20 degrees, and duration of 150–200 ms. A minimum of 20 horizontal head impulses was delivered randomly in the right and left directions. The mean vestibulo-ocular response (VOR) gains, which were automatically calculated, were used as parameters.

For RCT, sinusoidal stimulation was conducted in a dark room using the I-Portal NOTC-S system version 8.2 (NeuroKinetics, Pittsburgh, PA, USA). Several frequencies were tested, including 0.02 Hz, 0.04 Hz, 0.12 Hz, 0.25 Hz, 0.5 Hz, and 1.00 Hz. The gain was calculated by determining the ratio of the eye SPV to the chair velocity. For calibration, the patients sat on the rotary chair and their head was bent 30 degrees relative to the ground so that the lateral semicircular canal was parallel to the horizontal plane. Patients were instructed to follow a laser target for 6 s with their eyes in a completely dark room. The peak angular velocity was 60 degree/s in 0.12 Hz and 1.00 Hz stimulation. Gain was analyzed from 5 and 14 cycles in 0.12 Hz and 1.00 Hz stimulation, respectively. Sinusoidal stimulation for lateral canal gain in the dark condition was performed prior to the visual suppression (visual fixation) test.

Posturography (SMART EquiTest; Natus Medical Inc., Pleasanton, CA, USA before 21 December 2021; computerized dynamic posturography, Bertec, Columbus, OH, USA from 21 December 2021) was performed to test balance maintenance. The composite and vestibular scores on the sensory organization test of the CDP were calculated using the weighted average of the equilibrium scores.

The cervical and ocular vestibular-evoked myogenic potential (cVEMP and oVEMP) responses were recorded in the ipsilateral sternocleidomastoid muscle (cVEMP) or contralateral inferior oblique ocular muscle (oVEMP) using 95 dB HL and 500 Hz tone burst stimulation (ABaer, Natus Medical, Inc., Pleasanton, CA, USA). These tests were performed according to protocols used in previous reports [14].

The sub-normal vestibular function was defined the same as the laboratory diagnostic criteria of PVP (maximum peak slow-phase velocity on each side 6–25°/s in caloric test or lateral canal gain 0.6–0.8 bilaterally in vHIT or 0.12 Hz of sinusoidal harmonic stimulation gain 0.1–0.3 in RCT). All procedures and settings were consistent across the patients throughout the study period. Experienced medical technologists and audiologists performed the vestibular function tests.

### 2.3. Statistical Analysis

Pearson’s correlation analysis was used to investigate the relationships between VFT results, and partial correlation analysis was used to adjust for age. Univariate linear regression and third-order polynomial regression was used to investigate the relationship between VFT results and age. Pearson’s chi-squared test was used to evaluate the proportional significance. Analysis of variance and Tukey’s post hoc tests were used for multiple group comparisons. A *p*-value < 0.05 was considered to indicate statistical significance. Statistical analyses were conducted using SPSS software ver. 23 (IBM, Armonk, NY, USA).

## 3. Results

### 3.1. Information of Enrolled Subjects

The mean age of subjects was 65.19 years, and 70.7% were female (Table 1). As we enrolled subjects who underwent both the caloric test and vHIT, 100% of subjects underwent these tests. However, RCT, posturography, cervical VEMP (cVEMP), and ocular VEMP (oVEMP) were performed in 30.2%, 80.6%, 76.3%, and 62.0% of subjects, respectively. The mean values of the caloric test, vHIT, RCT, and posturography composite scores were within normal ranges. cVEMP was present in approximately 50% of subjects; however, oVEMP was present only in up to 14.8% of subjects.

The correlation matrix between the VFT results showed a strong correlation (R = 0.892 and 0.762 for the caloric SPV and vHIT, respectively) with the interaural test results (Table 2 and Table S1). Caloric SPV, RCT 0.12 Hz gain, and vHIT showed significant correlations with each other. Interestingly, the RCT 1.0 Hz gain showed a significant relationship with vHIT, but not with caloric SPV, which represents the lowest frequency stimulation. Posturography composite scores correlated only with the vHIT results. In addition, the vHIT showed significant correlations with all other VFT results.

### 3.2. Relation between VFT Results and Aging

**Figure 1:**
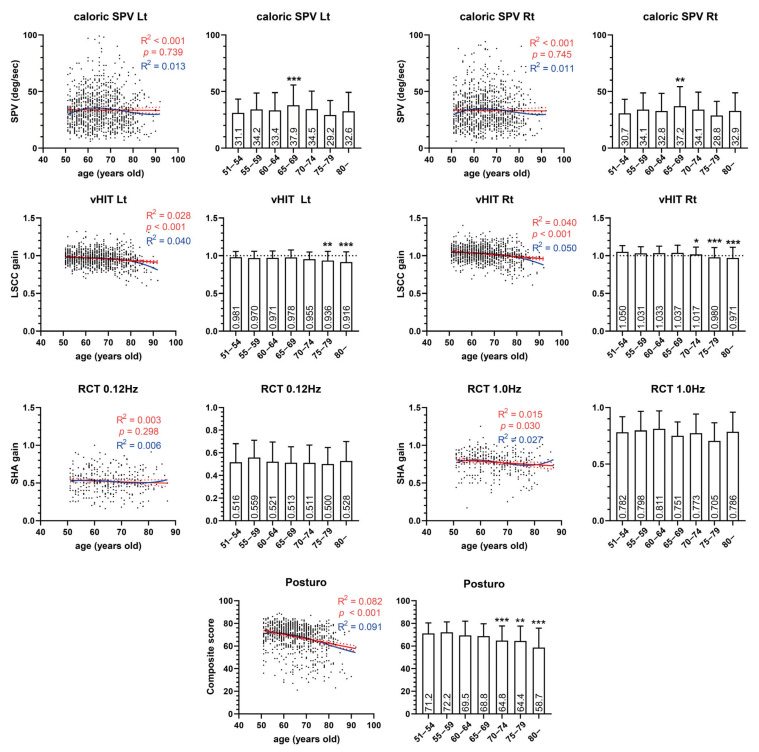
Dot plot and five-year bin graph of vestibular function test results and age. Each dot indicates individual result of subject. A red line indicates slope of best-fit line of linear regression, and red dotted bands indicate 95% confidence range. Blue curved line indicates the best-fit line of the third-order polynomial regression. Box and error bar indicate the average (number in the box) and standard deviation, respectively. SPV: slow-phase peak velocity, LSCC: lateral semi-circular canal, RCT: rotary chair test, Posturo: posturography composite score, SHA: sinusoidal harmonic acceleration test, R: correlation coefficient, *p*: *p*-value, *: *p* < 0.05, **: *p* < 0.01, ***: *p* < 0.001.

**Figure 2:**
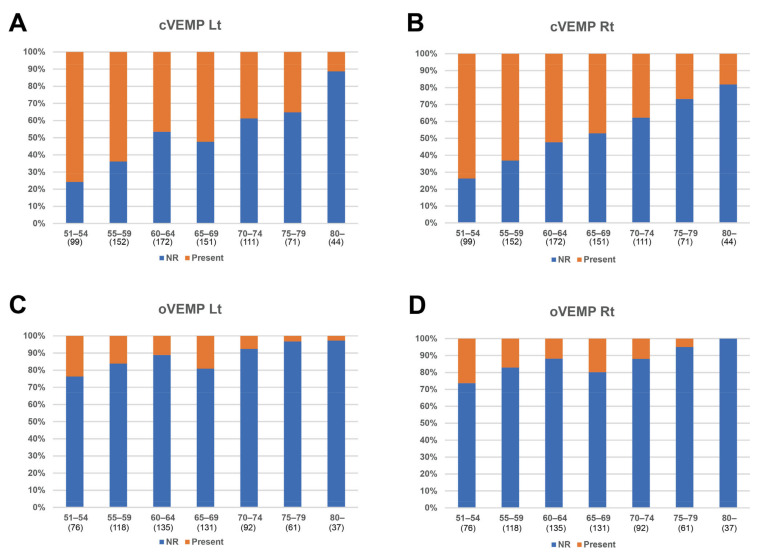
The presence rate of the vestibula evoked myogenic potential (VEMP) according to age. (**A**) Left cervical VEMP, (**B**) right cervical VEMP, (**C**) left ocular VEMP, and (**D**) right ocular VEMP. The Y-axis indicates rate of VEMP presence, and the X-axis indicates age. Number in parentheses is the number of subjects in the age group. NR: no response, Present: presence of VEMP response.

Next, the correlation between the VFT results and age was analyzed (Figure 1). Significant negative correlations were identified in the vHIT (*p* < 0.001), RCT 1.0 Hz gain (*p* = 0.030), and posturography composite scores (*p* < 0.001). However, the caloric test (*p* = 0.739 and 0.745 on the left and right sides, respectively) and RCT 0.12 Hz gain (*p* = 0.298) did not show a significant correlation with age. VEMPs, whose data were dichotomous, also showed a tendency for a decreased presence rate with aging (Figure 2). In subjects older than 60, fewer than half of subjects showed the presence of cVEMP.

### 3.3. Analysis of Groups Categorized by Age

**Figure 3:**
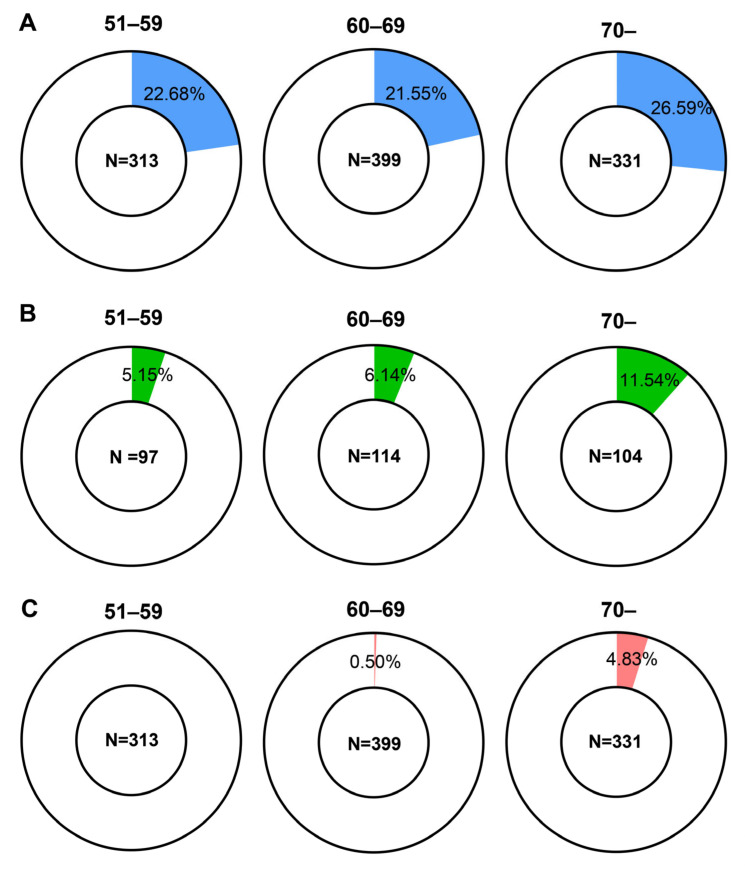
Proportion of sub-normal results (met the presbyvestibulopathy diagnostic criteria). Video head impulse test showed a significant (*p* < 0.001) difference between age groups, but other tests did not (*p* = 0.258 and 0.177 in caloric test and rotary chair test, respectively). (**A**) Blue area indicates the proportion of caloric test slow-phase peak velocity 6–20 °/s in both sides, (**B**) green area indicates the proportion of 0.12 Hz sinusoidal harmonic acceleration test gain 0.1–0.3, and (**C**) red area indicates the proportion of video head impulse test lateral canal gain 0.6–0.8 in both sides. *n*: number of subjects in the group.

Based on the diagnostic criteria for PVP, we investigated the proportion of patients who have sub-normal vestibular function, as defined by the PVP diagnostic criteria for laboratory tests. Group categorization was age 51–59 as the young group, age 60–69 as the elderly group, and age 70 as the very elderly group (Figure 3 and Table S2). Sub-normal caloric response (SPV 6–20°/s, in both sides) rates were 22.68%, 21.55%, and 26.59% in the 51–59, 60–69, and 70+ groups, respectively. The proportions of sub-normal caloric response were not significantly different among the groups (*p* = 0.258). In RCT 0.12 Hz (SHA test gain 0.1–0.3), sub-normal proportions were 5.15%, 6.14%, and 11.54% in each group, respectively, which was not significantly different between groups (*p* = 0.177). In vHIT (lateral canal gain 0.6–0.8, in both sides), 0%, 0.5%, and 4.83% showed sub-normal gain in each group, respectively, which was significantly different between groups (*p* < 0.001).

## 4. Discussion

In this study, the caloric response and RCT 0.12 Hz did not show a significant correlation with subject age. In contrast, vHIT and RCT 1.0 Hz showed significant correlations with age. This implies that aging is more likely to debilitate high-frequency VOR. As the frequency of VOR in response to ambient head movement ranges from 5 to 7 Hz, which is similar to that of vHIT [15,16,17], PVP symptoms can plausibly be explained by a decrease in high-frequency VOR, as represented by vHIT and RCT at 1.0 Hz. In contrast, the low-frequency VOR test, represented by the caloric test, did not decrease with age. In addition, VEMP and posturography, which previously were reported to be related to subject age, correlated well with age, as shown in previous studies [9,10,11,18].

Age-related decline in the gain in the vHIT has been previously reported by several groups. A study conducted in a small number of healthy volunteers found a distinctive decline in horizontal canal gain after 70 years of age [19]. This study also reported that the gain decline was prominent when the head impulse speed was high. Other previous studies have found a significant correlation between age and lateral canal gain, and a significant decline in gain in patients aged >70 or 80 years [20,21]. In our results, we also found a significant relationship (*p* < 0.001) between age and lateral canal gain in vHIT, as well as distinctively increased sub-normal population in subjects age >70 (4.83%) compared with other age groups (0% in the 51–59 age group and 0.5% in the 60–69 age group). In addition, a study by Janky et al. reported that elders having declined vHIT gain may not have balance complaints [22]. However, given that our results revealed a significant and exclusive correlation between vHIT and the posturography composite score, a decline in vHIT gain can affect balance function, regardless of subjective symptoms.

In the caloric test, there seems to be a lack of evidence that caloric response decreases with age. Instead, a study with a small group of healthy subjects aged 18–80 years revealed a positive relationship between the caloric SPV and age [23]. Several studies have reported that the caloric response peaks at 50–70 years of age, followed by a decline [24,25,26]. Several possible hypotheses have been suggested for this non-linear relationship between SPV and age; for instance, diminishing central inhibition followed by decreased vestibular function cancels the diminishing central inhibition [23,24]. Another possible reason is the morphologic change in the mastoid cavity with aging. A smaller mastoid air space has a higher caloric response, owing to its better thermal diffusivity [27,28]. Previous studies showed that mastoid pneumatization decreases with aging after peaking at the age of 20 [29,30]. Therefore, the caloric response can be increased with aging due to the shrinkage of mastoid air space before the degeneration of the vestibular system by aging. In addition, the distinguishable decrement in the caloric response may camouflage the increment in the response due to the shrinkage of the mastoid air space. Our results also indicated a tendency toward a declined caloric response in the age >70 group, although this was not found to have a significant relationship with age. Caloric SPV peaks in the age 65–69 group in our results (similar to previous studies) presented a non-linear relationship with age.

RCT has been reported to be independent of age but exceptionally high in children [7,23]. However, Chan et al. reported that the lowest frequency is the most sensitive to age-related VOR changes, which contradicts our results [7]. The difference from our study was that they recruited subjects with a wide range of ages, from 6 to more than 50. Since PVP can be diagnosed after the age of 60 years based on histologic evidence indicating the onset of vestibular organ degeneration at the age of 50, our study appears to more accurately reflect the relationship between age and RCT VOR in older individuals [21,31,32]. Our study found that higher frequency (1.0 Hz) SHA test in RCT is significantly related to age, but not lower frequency (0.12 Hz).

We did not conduct a statistical analysis between age and VEMP results because the VEMP data were dichotomous (present/absent). However, we found significant correlations between the presence of VEMP and age in the group analysis (Table S1). Our data are consistent with those of numerous studies on VEMP associated with aging [9,10,11]. However, as the PVP consensus document indicates, the VEMP results are somewhat variable from laboratory to laboratory, and too many elderly subjects were non-responders [4]. Furthermore, muscular atrophy due to aging can affect VEMP results [33].

The subgroup analysis of normal VFT patients and those with sub-normal vestibular function was conducted; the two groups consistently showed a correlation with age in vHIT and posturography (Figure S1). This suggests that aging leads to a degeneration in vestibular function in the general population, rather than indicating a specific disease for a particular group (PVP). The effect size of age on VFT results is very small, considering that R in Figure 1 was small in all tests, even though significant correlations were identified in several tests. The small probability of alpha error (*p*-value) despite the small effect size indicates that degeneration is highly variable but definite. In addition, the effect size might have been a bit underestimated due to the late onset (over 70) of aging degeneration of vestibular function when considering the result of vHIT.

Regarding PVP diagnosis, an issue can be raised regarding whether the caloric test and low-frequency RCT can accurately reflect the age-related decrease in the VOR, as they did not demonstrate significant correlations with age in elderly individuals. Nevertheless, the caloric test and low-frequency rotary chair testing are still useful tests that reflect the degeneration of vestibular function, regardless of its etiology. Therefore, excluding these tests from PVP diagnostic criteria because they are not correlated with age requires careful discussion. Contrarily, further research should be conducted to determine whether other test results (e.g., posturography and VEMP) provide sufficient evidence to be excluded from the criteria despite their distinct correlation with age.

This study has limitations. The enrolled subjects were not healthy volunteers, even though they did not have acute dizziness within 2 weeks before the tests. For instance, polyneuropathy due to diabetes or mild concussion without central signs could have been included. To compensate for this weakness, we applied strict exclusion criteria and included subjects who showed objectively normal VOR. However, because our exclusion criteria mainly focused on lateral canal tests (caloric, RCT, and vHIT) according to the diagnostic criteria of PVP, we could not ensure that all enrolled subjects had normal function in the otolith organs and other semicircular canals. In addition, we could not perform an analysis including hearing function because this is a retrospective study. It would be interesting to compare vestibular function and hearing function, which are structurally close, in a future study.

In conclusion, high-frequency stimulation tests such as the vHIT showed a significant negative correlation with age in elderly people. This suggests that high-frequency range vestibular function is vulnerable to aging, and this is more discernible in those aged over 70 years. The weak correlation between age and low-frequency vestibular function tests, such as the caloric test and low-frequency rotary chair testing, suggests the need to revisit the diagnostic criteria for PVP.

## Figures and Tables

**Table 1:** Information of enrolled subjects.

Factors	Value
Mean Age, years (SD)	65.19 (8.89)
Male:Female, *n* (%)	306 (29.3):737 (70.7)
Caloric test	
Mean Lt SPV, deg/sec (SD)	33.75 (15.50)
Mean Rt SPV, deg/sec (SD)	33.35 (15.25)
Mean CP, % (SD)	8.33 (5.41)
Number, *n* (%)	1043 (100)
vHIT, LSCC	
Mean Lt gain (SD)	0.964 (0.099)
Mean Rt gain (SD)	1.023 (0.104)
Number, *n* (%)	1043 (100)
RCT, SHA	
Mean 0.1 Hz gain (SD)	0.521 (0.157)
Mean 1.0 Hz gain (SD)	0.774 (0.156)
Number, *n* (%)	315 (30.2)
Posturography	
Mean Composite score (SD)	68.30 (11.94)
Number, *n* (%)	841 (80.6)
cVEMP	
Lt Present, *n* (%)	404 (50.5)
Lt Absent, *n* (%)	396 (49.5)
Rt Present, *n* (%)	399 (49.9)
Rt Absent, *n* (%)	401 (50.1)
Number, *n* (%)	800 (76.3)
oVEMP	
Lt Present, *n* (%)	87 (13.4)
Lt Absent, *n* (%)	563 (86.6)
Rt Present, *n* (%)	96 (14.8)
Rt Absent, *n* (%)	554 (85.2)
Number, *n* (%)	650 (62.0)
Total, *n* (%)	1043 (100)

SD: standard deviation, Lt: left, Rt: right, SPV: sum of slow-phase peak velocity in warm and cool stimulation, vHIT: video head impulse test, LSCC: lateral semicircular canal, RCT: rotary chair test, SHA: sinusoidal harmonic acceleration, cVEMP: cervical vestibular evoked myogenic potential, oVEMP: ocular vestibular evoked myogenic potential.

**Table 2:** Relationships between vestibular function tests.

R *p*-Value *n*	Caloric Lt	Caloric Rt	vHIT Lt	vHIT Rt	RCT 0.12	RCT 1.0	Posturo
Caloric Lt	1 N/A 1043						
Caloric Rt	0.892 *** <0.001 1043	1 N/A 1043					
vHIT Lt	0.163 *** <0.001 1043	0.165 *** <0.001 1043	1 N/A 1043				
vHIT Rt	0.115 *** <0.001 1043	0.115 *** <0.001 1043	0.762 *** <0.001 1043	1 N/A 1043			
RCT 0.12	0.225 *** <0.001 315	0.164 ** 0.004 315	0.149 ** 0.008 315	0.165 ** 0.003 315	1 N/A 315		
RCT 1.0	0.058 0.301 315	0.057 0.314 315	0.171 ** 0.002 315	0.188 ** 0.001 315	0.438 *** <0.001 315	1 N/A 315	
Posturo	0.028 0.413 841	0.024 0.490 841	0.136 *** <0.001 841	0.123 *** <0.001 841	−0.049 0.422 276	0.058 0.335 276	1 N/A 841

R: Correlation coefficient, *n*: number of subjects included in analysis, Lt: left, Rt: right, Caloric: sum of slow-phase peak velocity in warm and cool stimulation, vHIT: lateral semicircular canal gain in video head impulse test, RCT: gain of sinusoidal harmonic acceleration in rotary chair test, Posturo: composite score in posturography. **: *p* < 0.01, ***: *p* < 0.001, N/A: not applicable.

## Data Availability

The data are available from the corresponding author upon reasonable request.

## Supplementary Materials

The following supporting information can be downloaded at https://www.mdpi.com/article/10.3390/diagnostics14121224/s1. Table S1: Relationships between vestibular function tests (age adjusted). Table S2: Vestibular function test results of three age groups. Figure S1: The subgroup analysis of normal VFT patients and those with subnormal vestibular function.

## References

[1] Graafmans W.C., Ooms M.E., Hofstee H.M.W., Bezemer P.D., Bouter L.M., Lips P. (1996). Falls in the elderly: A prospective study of risk factors and risk profiles. Am. J. Epidemiol..

[2] Tinetti M.E., Williams C.S., Gill T.M. (2000). Dizziness among older adults: A possible geriatric syndrome. Ann. Intern. Med..

[3] Iwasaki S., Yamasoba T. (2015). Dizziness and Imbalance in the Elderly: Age-related Decline in the Vestibular System. Aging Dis..

[4] Agrawal Y., Van de Berg R., Wuyts F., Walther L., Magnusson M., Oh E., Sharpe M., Strupp M. (2019). Presbyvestibulopathy: Diagnostic criteria Consensus document of the classification committee of the Barany Society. J. Vestibul. Res. Equil..

[5] Lee S.U., Park S.H., Kim H.J., Koo J.W., Kim J.S. (2016). Normal Caloric Responses during Acute Phase of Vestibular Neuritis. J. Clin. Neurol..

[6] Halmagyi G.M., Curthoys I.S., Cremer P.D., Henderson C.J., Todd M.J., Staples M.J., Dcruz D.M. (1990). The Human Horizontal Vestibuloocular Reflex in Response to High-Acceleration Stimulation before and after Unilateral Vestibular Neurectomy. Exp. Brain Res..

[7] Chan F.M., Galatioto J., Amato M., Kim A.H. (2016). Normative data for rotational chair stratified by age. Laryngoscope.

[8] Zuniga S.A., Adams M.E. (2021). Efficient Use of Vestibular Testing. Otolaryngol. Clin. N. Am..

[9] Su H.C., Huang T.W., Young Y.H., Cheng P.W. (2004). Aging effect on vestibular evoked myogenic potential. Otol. Neurotol..

[10] Janky K.L., Shepard N. (2009). Vestibular evoked myogenic potential (VEMP) testing: Normative threshold response curves and effects of age. J. Am. Acad. Audiol..

[11] Ochi K., Ohashi T. (2003). Age-related changes in the vestibular-evoked myogenic potentials. Otolaryngol. Head Neck Surg..

[12] You S.H. (2005). Joint position sense in elderly fallers: A preliminary investigation of the validity and reliability of the SENSERite measure. Arch. Phys. Med. Rehabil..

[13] Jongkees L.B., Maas J.P., Philipszoon A.J. (1962). Clinical nystagmography. A detailed study of electro-nystagmography in 341 patients with vertigo. Pract. Otorhinolaryngol..

[14] Jeong J., Jung J., Lee J.M., Suh M.J., Kwak S.H., Kim S.H. (2017). Effects of Saccular Function on Recovery of Subjective Dizziness After Vestibular Rehabilitation. Otol. Neurotol..

[15] Grossman G.E., Leigh R.J., Abel L.A., Lanska D.J., Thurston S.E. (1988). Frequency and velocity of rotational head perturbations during locomotion. Exp. Brain Res..

[16] Hirasaki E., Moore S.T., Raphan T., Cohen B. (1999). Effects of walking velocity on vertical head and body movements during locomotion. Exp. Brain Res..

[17] Halmagyi G.M., Chen L., MacDougall H.G., Weber K.P., McGarvie L.A., Curthoys I.S. (2017). The Video Head Impulse Test. Front. Neurol..

[18] Eriksen N.D., Hougaard D.D. (2023). Age- and gender-specific normative data on computerized dynamic posturography in a cohort of Danish adults. Eur. Arch. Otorhinolaryngol..

[19] Matino-Soler E., Esteller-More E., Martin-Sanchez J.C., Martinez-Sanchez J.M., Perez-Fernandez N. (2015). Normative data on angular vestibulo-ocular responses in the yaw axis measured using the video head impulse test. Otol. Neurotol..

[20] Kim T.H., Kim M.B. (2018). Effect of aging and direction of impulse in video head impulse test. Laryngoscope.

[21] Li C., Layman A.J., Geary R., Anson E., Carey J.P., Ferrucci L., Agrawal Y. (2015). Epidemiology of vestibulo-ocular reflex function: Data from the Baltimore Longitudinal Study of Aging. Otol. Neurotol..

[22] Janky K.L., Patterson J.N., Shepard N.T., Thomas M.L.A., Honaker J.A. (2017). Effects of Device on Video Head Impulse Test (vHIT) Gain. J. Am. Acad. Audiol..

[23] Maes L., Dhooge I., D’Haenens W., Bockstael A., Keppler H., Philips B., Swinnen F., Vinck B.M. (2010). The effect of age on the sinusoidal harmonic acceleration test, pseudorandom rotation test, velocity step test, caloric test, and vestibular-evoked myogenic potential test. Ear Hear..

[24] Bruner A., Norris T.W. (1971). Age-related changes in caloric nystagmus. Acta Otolaryngol. Suppl..

[25] Mulch G., Petermann W. (1979). Influence of age on results of vestibular function tests. Review of literature and presentation of caloric test results. Ann. Otol. Rhinol. Laryngol. Suppl..

[26] Karlsen E.A., Hassanein R.M., Goetzinger C.P. (1981). The effects of age, sex, hearing loss and water temperature on caloric nystagmus. Laryngoscope.

[27] Zangemeister W.H., Bock O. (1979). The influence of pneumatization of mastoid bone on caloric nystagmus response. A clinical study and a mathematical model. Acta Otolaryngol..

[28] Patki A.U., Ronen O., Kaylie D.M., Frank-Ito D.O., Piker E.G. (2016). Anatomic Variations in Temporal Bones Affect the Intensity of Nystagmus During Warm Caloric Irrigation. Otol. Neurotol..

[29] Lee D.H., Jun B.C., Kim D.G., Jung M.K., Yeo S.W. (2005). Volume variation of mastoid pneumatization in different age groups: A study by three-dimensional reconstruction based on computed tomography images. Surg. Radiol. Anat..

[30] Chatterjee D., Ghosh T.B., Ghosh B.B. (1990). Size Variation of Mastoid Air Cell System in Indian People at Different Age-Groups—A Radiographic Planimetric Study. J. Laryngol. Otol..

[31] Kristinsdottir E.K., Nordell E., Jarnlo G.B., Tjader A., Thorngren K.G., Magnusson M. (2001). Observation of vestibular asymmetry in a majority of patients over 50 years with fall-related wrist fractures. Acta Otolaryngol..

[32] Lin H.W., Bhattacharyya N. (2012). Balance disorders in the elderly: Epidemiology and functional impact. Laryngoscope.

[33] Dabiri S., Yazdani N., Esfahani M., Tari N., Adil S., Mahvi Z., Rezazadeh N. (2017). Analysis of Saccular Function with Vestibular Evoked Myogenic Potential Test in Meniere’s Disease. Acta Med. Iran..

