# Supplementary material for: High-Frequency Vestibular Function Is Vulnerable to Presbyvestibulopathy
Source: Diagnostics (Basel). 2024 Jun 11;14(12):1224. doi: 10.3390/diagnostics14121224 (PMC11202626; doi:10.3390/diagnostics14121224)

Supplementary Table 1. Relationships between vestibular function tests. (Age adjusted)

R<br>p-value<br>df	Caloric Lt	Caloric Rt	vHIT Lt	vHIT Rt	RCT 0.12	RCT 1.0	Posturo
Caloric Lt	1<br>N/A<br>0						
Caloric Rt	.892***<br><.001<br>1040	1<br>N/A<br>0					
vHIT Lt	.164***<br><.001<br>1040	.166***<br><.001<br>1040	1<br>N/A<br>0				
vHIT Rt	.115***<br><.001<br>1040	.115***<br><.001<br>1040	.754***<br><.001<br>1040	1<br>N/A<br>0			
RCT 0.12	.225***<br><.001<br>312	.164**<br>.004<br>312	.141**<br>.012<br>312	.157**<br>.005<br>312	1<br>N/A<br>0		
RCT 1.0	.058<br>.308<br>312	.056<br>.321<br>321	.154**<br>.006<br>312	.169**<br>.003<br>312	.435***<br><.001<br>312	1<br>N/A<br>0	
Posturo	.026<br>.444<br>838	.022<br>.527<br>838	.093**<br>.007<br>838	.069*<br>.045<br>838	-.068<br>.260<br>273	.025<br>.684<br>273	1<br>N/A<br>0

R: Partial correlation coefficient, df: degree of freedon, Lt: left, Rt: right, Caloric: sum of slow phase peak velocity in warm and cool stimulation, vHIT: lateral semicircular canal gain in video head impulse test, RCT: gain of sinusoidal harmonic acceleration in rotary chair test, Posturo: composite score in posturography. *: $p < 0.05$, **: $p < 0.01$, ***: $p < 0.001$, N/A: not applicable.

Supplementary Table 2. Vestibular function test results of three age groups.

	51-59	60-69	70-	p-value
Caloric test				
Lt SPV, deg/sec (SD)	32.95 (13.68)	35.47 (16.83)[a]	32.44 (15.29)[a]	0.017*
Rt SPV, deg/sec (SD)	32.72 (13.92)	34.82 (16.45)	32.18 (14.84)	0.045*
CP, % (SD)	7.93 (5.32)	8.17 (5.34)	8.91 (5.56)	0.052
Number, n (% from group)	313 (100)	399 (100)	331 (100)	
vHIT, LSCC				
Lt gain (SD)	0.974 (0.083)[a]	0.974 (0.094)[b]	0.940 (0.113)[ab]	<0.001*
Rt gain (SD)	1.039 (0.088)[a]	1.035 (0.098)[b]	0.995 (0.120)[ab]	<0.001*
Number, n (% from group)	313 (100)	399 (100)	331 (100)	
RCT, SHA				
0.1 Hz gain (SD)	0.539 (0.158)	0.516 (0.159)	0.509 (0.155)	0.367
1.0 Hz gain (SD)	0.790 (0.154)	0.783 (0.146)	0.749 (0.168)	0.127
Number, n (% from group)	97 (31.0)	114 (28.6)	104 (31.4)	
Posturography				
Composite score (SD)	71.80 (9.22)[ab]	69.36 (11.21)[bc]	63.66 (13.58)[ac]	<0.001*
Number, n (% from group)	248 (79.2)	330 (82.7)	263 (79.5)	
cVEMP				
Lt Present, n (%)	172 (68.5)	159 (49.2)	73 (32.3)	<0.001†
Rt Present, n (%)	169 (67.3)	161 (49.8)	69 (30.5)	<0.001†
Number, n (% from group)	251 (80.2)	323 (81.0)	226 (68.3)	
oVEMP				
Lt Present, n (%)	37 (19.1)	40 (15.0)	10 (5.3)	<0.001†
Rt Present, n (%)	40 (20.6)	42 (15.8)	14 (7.4)	0.001†
Number, n (% from group)	194 (62.0)	266 (66.7)	190 (57.4)	
Number, n (% from total)	313 (30.0)	399 (38.3)	331 (31.7)	N/A

SD: standard deviation, Lt: left, Rt: right, SPV: sum of slow phase peak velocity in warm and cool stimulation, vHIT: video head impulse test, LSCC: lateral semicircular canal, RCT: rotary chair test, SHA: sinusoidal harmonic acceleration, cVEMP: cervical vestibular evoked myogenic potential, oVEMP: ocular vestibular evoked myogenic potential. *: significant result in analysis of variance test. †: significant result in Pearson's chi-square test. [a,b,c]: significant results in post-hoc Tukey test.

Supplementary Figure 1.

The normal vestibular function group indicates normal range results in the video head impulse test, caloric test, and rotary chair test. The sub-normal vestibular function group indicates at least one abnormal result in any of the three tests. (A) Video head impulse test gain of the left lateral semicircular canal in the normal vestibular function group. (B) Video head impulse test gain of the left lateral semicircular canal in the sub-normal vestibular function group. (C) Posturography composite score of the normal vestibular function group. (D) Posturography composite score of the sub-normal vestibular function group. vHIT: video head impulse test, Lt: left, R: correlation coefficient, p: p-value, posturo: posturography.

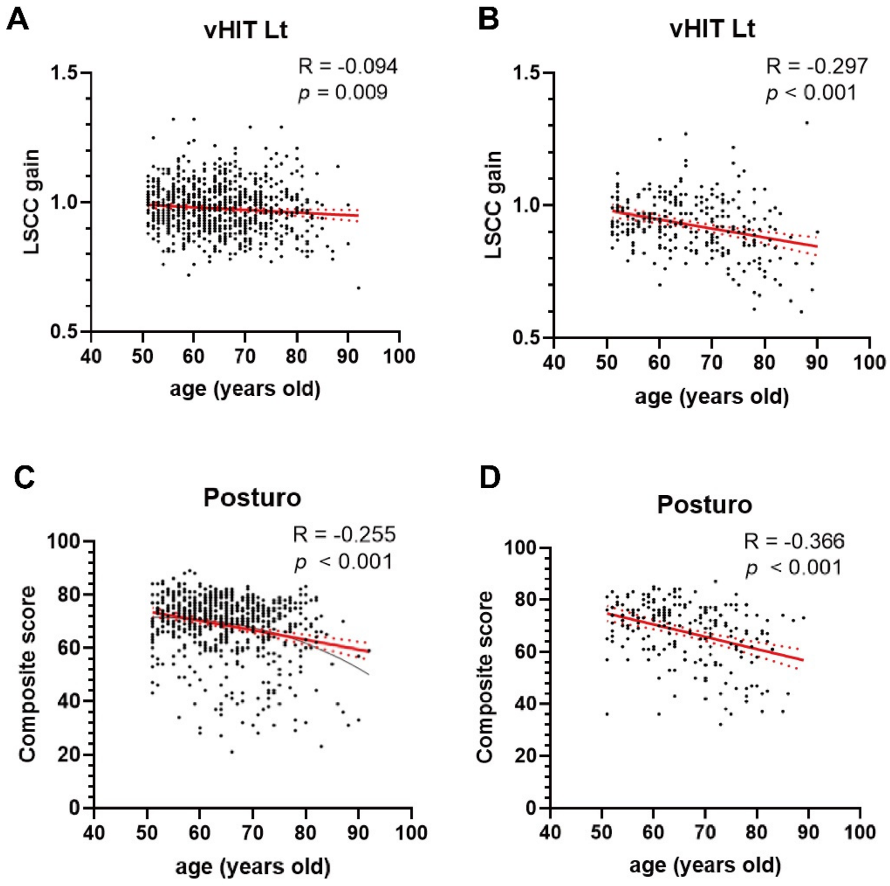

Supplement: Supplementary file 1 [file diagnostics-14-01224-s001.zip › diagnostics-3025079-supplementary.pdf]
